# Supplementary material for: The effects of substance use on non-communicable diseases among older adults aged 60 and above in the North-eastern States of India
Source: PLoS One. 2024 Sep 10;19(9):e0307603. doi: 10.1371/journal.pone.0307603 (PMC11386429; doi:10.1371/journal.pone.0307603)
Supplement: Attachment — Submitted filename: Response to Reviewers.docx [file pone.0307603.s002.docx]

Reviewer 1:

Abstract:

Objective: To assess the prevalence of NCDs and strength of association of substance abuse among geriatric population of Northeastern states in India, for the development of preventive strategies, but not for implementation.

Methods:

Data received from the 'Longitudinal Ageing Study in India' (LASI -wave 1 2017-18) for the present communication (paper), not for the study.

Conclusion:

Since, high prevalence of hypertension correlated with the high level of substance abuse, require immediate attention to develop an appropriate intervention strategies for its control (substance abuse) and prevention of hypertension.

Response:
Yes, we have incorporated the changes in the abstract as has been advised.
