# Supplementary material for: The effects of substance use on non-communicable diseases among older adults aged 60 and above in the North-eastern States of India
Source: PLoS One. 2024 Sep 10;19(9):e0307603. doi: 10.1371/journal.pone.0307603 (PMC11386429; doi:10.1371/journal.pone.0307603)
Supplement: Attachment — Submitted filename: Response to Reviewers.docx [file pone.0307603.s004.docx]

Editor:

There is need for a thorough language (English) editing of the manuscript. Please refer to some of the comments, highlighted texts (in yellow colour), and edits (in track change mode) given in the attached copy of your revised manuscript. 

Response:
Yes, we have incorporated the changes in the manuscript as editor has advised.
